# Sociodemographic and Psychological Risk Factors for Anxiety and Depression: Findings from the Covid-19 Health and Adherence Research in Scotland on Mental Health (CHARIS-MH) Cross-sectional Survey

**DOI:** 10.1007/s12529-021-09967-z

**Published:** 2021-03-03

**Authors:** Gill Hubbard, Chantal den Daas, Marie Johnston, Diane Dixon

**Affiliations:** 1 Health Psychology Group, University of Aberdeen Institute of Applied Health Sciences, Aberdeen, Scotland; 2 Department of Nursing and Midwifery, University of the Highlands and Islands, Institute of Health Research and Innovation, Inverness, Scotland

**Keywords:** Coronavirus, COVID-19, Public mental health, Loneliness, Social support, Threat perception, Illness representations

## Abstract

**Background:**

Investigations about mental health report prevalence rates with fewer studies investigating psychological and social factors influencing mental health during the Covid-19 pandemic. Study aims: (1) identify sociodemographic groups of the adult population at risk of anxiety and depression and (2) determine if the following social and psychological risk factors for poor mental health moderated these direct sociodemographic effects: loneliness, social support, threat perception, illness representations.

**Methods:**

Cross-sectional nationally representative telephone survey in Scotland in June 2020. If available, validated instruments were used, for example, Patient Health Questionnaire (PHQ-4) to measure anxiety and depression. Simple linear regressions followed by examination of moderation effect.

**Results:**

A total of 1006 participants; median age 53 years, 61.4% female, from all levels of area deprivation (i.e., 3.8% in the most deprived decile and 15.6% in the most affluent decile). Analyses show associations of anxiety and depression with sociodemographic (age, gender, deprivation), social (social support, loneliness) and psychological factors (perceived threat and illness representations). Mental health was poorer in younger adults, women and people living in the most deprived areas. Age effects were exacerbated by loneliness and illness representations, gender effects by loneliness and illness representations and deprivation effects by loneliness, social support, illness representations and perceived threat. In each case, the moderating variables amplified the detrimental effects of the sociodemographic factors.

**Conclusions:**

These findings confirm the results of pre-Covid-19 pandemic studies about associations between sociodemographics and mental health. Loneliness, lack of social support and thoughts about Covid-19 exacerbated these effects and offer pointers for pre-emptive action.

## Introduction

### Groups at Risk of Poor Mental Health During Covid-19 Pandemic

The Covid-19 pandemic represents a threat to mental health. It is a threat to clinical populations and clinicians. Studies conducted in previous epidemics (SARs-CoV in 29 countries in 2002–2004, Ebola virus disease in West Africa in 2014–2016, Middle East Respiratory Syndrome in South Korea in 2015) suggest that people diagnosed and treated for Covid-19 will experience both immediate and longer-term mental health problems [1]. A systematic review and meta-analysis of studies about healthcare workers during the current Covid-19 pandemic show a prevalence rate of 23.2% for anxiety and 22.8% for depression [2], which could have long-term psychological implications [3]. Covid-19 is also a threat to the general population. A systematic review and meta-analysis of international evidence report prevalence for stress, anxiety and depression as 29.6%, 31.9% and 33.7%, respectively [4]. In a large (*n* = 53,351) United Kingdom (UK) repeated cross-sectional survey, mental health, assessed using the 12-item General Health Questionnaire, found that population prevalence of clinically significant levels of mental distress rose from 18.9% in 2018–2019 to 27.3% in April, 2020, 1 month into UK lockdown, thereby suggesting that the Covid-19 pandemic led to a deterioration of mental health [5]. Similarly, another UK online survey found that 40% of respondents reported feeling more anxious during than before Covid-19, and this was severe in 8% of cases [6].

Cross-sectional surveys conducted in the UK in the early months (March–July) of the pandemic highlight groups of the population who appear most at risk of poor mental health [6–14]. They show women to be more anxious than men (69% vs. 52% [7]; 27% vs. 18% [9]; 26% vs. 18% [14]), people living in deprived than more affluent areas (28% vs. 20% [14]) and younger people (25% 18–29-year olds reporting no anxiety in the last 2 weeks vs. ~ 78% of people aged 80+ [6]). Another UK survey found mental well-being to be worse in people with physical multimorbidity (OR = 2.35, 95%CI = 1.61–3.46) [12]. The international literature also shows that these groups are especially vulnerable to experiencing poor mental health during the Covid-19 pandemic [15–20], although not all studies show statistically significant differences, and other groups of the population have also been identified as at risk of poor mental health such as pregnant women, migrant workers and people who are homeless [21]. This body of work therefore consistently shows that women, younger adults and people living in deprived areas are most at risk of poor mental health but it is limited in its ability to provide explanations for why these people are most at risk during the current Covid-19 pandemic. Hence, understanding the extent to which Covid-19-specific factors such as restrictions on accessing social support, perceived threat and beliefs about the illness Covid-19 exacerbate gender, age and area deprivation effects on mental health is important.

### Social and Psychological Risk Factors for Poor Mental Health that Might Be Exacerbated During the Covid-19 Pandemic

Risk factors for poor mental health in adulthood are diverse and range from genetic and biomedical to psychological and sociocultural [22–24]. A small number of social and psychological risk factors have recently been studied as having a direct, moderating or mediating effect on mental health during the Covid-19 pandemic. This body of work contributes towards understanding why there are variations in mental health during the Covid-19 pandemic.

#### Loneliness and Social Support

Recently studied social risk factors are the inter-related concepts of loneliness (the subjective feeling state of being alone, or apart from others), social isolation (objective physical separation from other people) and social support (objective degree to which one is socially connected and the subjective perception of the availability of support from others) [23, 25]. The structural characteristics of social relationships include the number and type of people with whom a person interacts including relationship status (married, living together, divorced etc.) whereas the functional characteristics of social relationships include the purpose and nature of relationships [26]. The number, type and function of social relationships are deemed relevant to the Covid-19 pandemic due to social distancing measures, the reduction in face-to-face opportunities to socialise and connect with family, neighbours and friends and temporary closure of places where people gather (e.g. workplace, shopping centre, places of worship, galleries) [27]. A stakeholder survey of people with lived experience of mental health problems and their supporters, and a nationally representative general population survey, both carried out in March 2020 in the UK, reported the importance of keeping in regular contact with friends and family, often online, as a key factor in maintaining mental health and well-being [11]. Social interaction during the pandemic appears to vary between different groups of the UK population. A survey carried out in the first few months of the pandemic in Wales found that 39% of people living in affluent areas were communicating with neighbours compared with 27% of people living in deprived areas, 15% often felt isolated and 7% felt lonely [14]. A UK survey found that 47% of adults reported that communicating with friends/family was harder than before the Coronavirus outbreak, with women in particular finding it ‘much harder’ than men (25% vs. 19%) [13]. The UK Opinions and Lifestyle Survey found that around 18% of adults experienced feeling lonely some of the time and 6% often or always in May 2020 [7]. Another study conducted between March and July 2020 of over 70,000 people indicates that people who felt most lonely prior to Covid-19 in the UK now have even higher levels of loneliness [28]. A survey conducted in Germany in the early stages of the pandemic found that social contact with people was negatively correlated with self-reported anxiety [18]. In sum, these social factors, which are generally protective of mental health, may be impaired by restrictions imposed to manage the pandemic and may exacerbate the sociodemographic differences in levels of mental health during the pandemic.

#### Perceived Threat

A psychological risk factor for poor mental health during the Covid-19 pandemic is perceived threat (may also be referred to as risk perception). A Chinese study conducted by Wang and colleagues, using the Depression, Anxiety and Stress Scale to measure anxiety and stress, found that low perceived likelihood of contracting Covid-19 was significantly associated with a low anxiety score, (*B* = −0.36, 95% CI: −0.63 to −0.09) and low perceived likelihood of surviving Covid-19 if infected was significantly associated with a high stress score (*B* = 0.34, 95% CI: 0.01 to 0.68) [19]. Studies conducted in China during the Covid-19 pandemic found that higher perceived susceptibility and severity and impact were strong predictors of higher levels of both state and trait anxiety [29] and that threat perception was associated with depression [30]. A study exploring the relationship between social support, threat perception (defined as probability of having Covid-19, control over getting infected, concern about being put at risk) and anxiety in pregnant women in China during the Covid-19 pandemic found that threat perception was negatively correlated with social support, and positively correlated with anxiety and that social support was negatively correlated with anxiety [31]. The study also found that threat perception mediated the relationship between social support and anxiety [31]. It is conceivable therefore that perceived threat of Covid-19 may exacerbate the sociodemographic differences in levels of mental health during the pandemic. Based on this empirical evidence, Protection Motivation Theory, which contends that people make a threat appraisal based on how severe they believe the threat is and how vulnerable they perceive themselves to be to the threat, may be useful for understanding mental health during the Covid-19 pandemic [32].

#### Illness Representation

Illness perceptions are associated with anxiety and depression across a range of chronic conditions [33–36] and alongside loneliness and social support and perceived threat, may also exacerbate the sociodemographic differences in levels of mental health during the pandemic. Leventhal’s common-sense model of self-regulation (CS-SRM) [37] proposes that people construct representations of a health threat, which help them make sense of their experiences and provide a basis for their own coping responses. Beliefs about illness are central to the model and incorporate five key components: beliefs about the nature (identity), time-course (timeline), personal impact (consequences), causal factors (cause) and feasibility of control or cure (control/cure) of the illness [37].

Thus, there is evidence that both social and psychological factors may be related to mental health, both in general and during the Covid-19 pandemic. We therefore aimed to examine whether these specific Covid-19-related psychological and social factors modify the sociodemographic associations with mental health, and potentially offer a route to successful interventions during the current and future pandemics since these factors can be potentially modified.

### Aims and Hypothesises

The aims of the Covid-19 Health and Adherence Research in Scotland (CHARIS) project [38] were to investigate mental health in the adult population during the Covid-19 pandemic and to explain variations in mental health. Hence, one of the CHARIS studies (CHARIS-MH) was about mental health and was conducted when Scotland was just moving out of lockdown to phase 1 of the Government’s route map out of lockdown [39]. Key phase 1 changes included being able to meet up with another household outdoors in small numbers, thereby possibly ameliorating people’s experiences of loneliness and social support. The aims of the study were to (1) confirm sociodemographic groups of the adult population at risk of poor mental health during the Covid-19 pandemic and (2) determine if the following social and psychological risk factors for poor mental health moderated these direct effects during the Covid-19 pandemic: loneliness, social support, threat perception and illness representation.

**Fig. 1:**
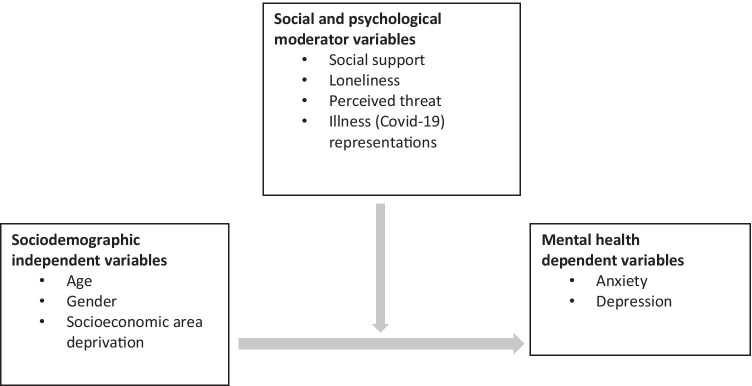
Conceptual model

We hypothesised (1) that age, gender and socioeconomic area deprivation would have direct effects on self-reported anxiety and depression also during the Covid-19 pandemic. Specifically, women, younger adults and people living in deprived areas have worse mental health and (2) that the association between these variables would be influenced by social and psychological risk factors for poor mental health that might have been exacerbated by the Covid-19 pandemic (see Fig. 1). Specifically, we hypothesised that more loneliness, less social support, higher threat perception and more negative illness representations would be associated with worse mental health. We explored whether these risk factors modify the relationship between age, gender and socioeconomic area deprivation and mental health. While these sociodemographic groups are not modifiable, this is information about who to target to reduce the effect of Covid-19 on mental health. If the social and psychological risk factors (loneliness, social support, threat perception, illness representation) exacerbate and moderate these relationships, these risk factors are potentially modifiable in interventions. Hence, this study may help governments and other health agencies target those groups of the population at risk of poor mental health and tailor interventions to address key risk factors. The study is likely to make a unique contribution to the emerging body of international work about mental health during the Covid-19 pandemic as follows: (1) it draws on theorised social and psychological constructs (loneliness and social support, threat perception and illness representation) to understand mental health during the Covid-19 pandemic and is relatively novel by the inclusion of illness representations. Use of these higher-order constructs means that the study is able to go beyond describing the state of mental health at population level by providing theorised explanations for the observed variations in mental health, (2) it is one of the few studies that includes a measure of socioeconomic area deprivation (alongside age and gender) which is a crucial factor for understanding population level variations in mental health [40], (3) it assesses mental health and potentially modifiable social and psychological risk factors at a critical period in the Covid-19 crisis, which is just when lockdown restrictions were being lifted, (4) it is a nationally representative survey in Scotland and one of the few studies that administers the survey by telephone rather than the web and therefore minimises the exclusion of people in deprived communities and in particular older people, and those with no educational qualifications, including those with poor literacy [41]. The results of the study can be used by governments to target interventions at those groups of the population with worse mental health and moreover, address through intervention those social and psychological factors that are likely to exacerbate mental health problems during the current and future pandemics.

## Method

The protocol for the CHARIS project is published [38]. Below is a description of the design and methods for this particular CHARIS study about mental health (CHARIS-MH).

### Design and Setting

A serial weekly nationally representative cross-sectional observational study aiming to recruit approximately 500 randomly selected adults in Scotland in the first 2 weeks of June 2020. The survey was administered by telephone in order to minimise the exclusion of people in deprived communities and in particular older people, and those with no or few educational qualifications, including those with poor health literacy [41]. Further, the telephone method is important in studies about mental health given that there is some evidence to suggest that Internet users in deprived areas are less likely to report feeling lonely and have higher mental well-being scores [41].

### Participants

Scotland has a total population of 5.4 million, 83% of whom are adults. All adult men and women aged 16 or older, able to speak English, and currently living in Scotland were eligible to participate. No other exclusion criteria were applied (people with all possible previous mental health states could be included). CHARIS was administered by a commercial polling company (Ipsos MORI Scotland) who sampled participants using random digit dialling to landlines and targeted mobiles. Quotas were applied to ensure that a representative sample of Scotland adults was achieved. Quotas were based on gender (52% female), age, working status (42% working fulltime) and geographical locations (distribution over the Scottish Parliament regions with a leeway of 30% for feasibility [38]). Participants did not receive compensation for their participation.

### Variables

#### Dependent Variables

Two mental health-dependent variables were measured. Anxiety and depression were measured using the 4-item Patient Health Questionnaire (PHQ-4), which is an ultra-brief screening scale for anxiety and depression [42]. The introductory text was slightly adapted for a telephone as opposed to written administered survey. Participants were asked: 'Over the last 2 weeks, how often have you been bothered by the following problems? Tell me which answer option best applies: (1) feeling nervous, anxious or on edge; (2) not being able to stop or control worrying; (3) feeling down, depressed or hopeless; (4) little interest or pleasure in doing things'. For each item, participants were given the following response options: not at all, several days, more than half of the days and nearly every day. The total score ranges from 0 to 12 with categories of psychological distress being none 0–2, mild 3–5, moderate 6–8 and severe 9–12. The anxiety subscale is the sum of items 1 and 2 and the depression subscale is the sum of items 3 and 4. On each subscale, a score of 3 or greater is considered positive for screening for anxiety and depression purposes [42].

#### Independent Variables

Three sociodemographic variables predictive of mental health were assessed. Age was assessed continuously in years and gender was assessed using Office for National Statistics binary categories (0 = female, 1 = male) [43]. Socioeconomic area deprivation was assessed using the Scottish Index of Multiple Deprivation (SIMD), which looks at the extent to which an area is deprived across seven domains: income, employment, education, health, access to services, crime and housing [44]. All 6976 data zones, postcodes, were grouped into 10 bands (deciles), each containing 10% of the data zones. Decile 1 contains the 10% most deprived data zones in Scotland, decile 10 contains the 10% least deprived data zones in Scotland. The deciles were used as a continuous variable in the analyses.

#### Moderator Variables

Five moderator variables were measured.

Social support was measured in two ways; first, by adapting 5 items from the ENRICHD Social Support Instrument [45]: ‘To what extent if at all, do you agree or disagree with each of the following statements. You have someone….(1) you can count on to listen to you when you need to talk, (2) to give you good advice about a problem, (3) who shows you love and affection, (4) to help you with daily chores, (5) Do you have as much contact as you would like with someone you feel close to, someone in whom you can trust and confide?’ There were four responses ranging from ‘strongly agree’ (score 4) to ‘strongly disagree’ (score 1), and with the option of responding ‘don’t know’ or ‘prefer not to say.’ We calculated an average score with the 4 items scored (1 to 4) and the contact question (yes = 4, no = 1), where a low score reflected low social support and a high score high social support. The second way social support was measured was by relationship status using the following six categories: married/civil partnership, living together, single, widowed, divorced and separated. We recoded the six categories into two: married/living together, reflecting high social support, and the other categories (single, widowed, divorced or separated) reflecting low social support.

Loneliness was measured using the following three-item loneliness scale [46]: ‘The next question is about how you feel about different aspects of your life. For each aspect, please tell me how often, if at all, you feel that way. How often, do you feel…? (1) that you lack companionship, (2) left out, (3) isolated from others’? There were five response options ranging from ‘always’ to ‘never’ and with the option of responding ‘don’t know’ or ‘prefer not to say.’ We calculated an average of the 3 items (1 = never lonely, 5 = always lonely).

Perceived threat was measured using two items to assess the constructs perceived severity and perceived vulnerability: ‘If you were ill with Covid-19 it would be serious for you;’ and ‘It is likely that you will get Covid-19’. There were four responses ranging from ‘strongly agree’ to ‘strongly disagree’ and with the option of responding ‘don’t know’ or ‘prefer not to say.’ In line with the protection motivation theory, we multiplied the measures of perceived severity (scale 1–4) and vulnerability (scale 1–4), to produce a perceived threat score (range 1–16) [38]. The composite score indicates the combined threat of these two distinct dimensions of threat perception.

Illness (Covid-19) representation was measured using an adapted brief illness perception questionnaire [47]. The brief illness perception questionnaire uses a single statement to assess each of the constructs from CS-SRM, namely: identity, consequences, duration (time-line), recurrence (time-line), personal control, treatment contro, and emotional representation (worried and anxious); participants indicate their level of agreement with each statement using a four-point Likert rating scale. Questions were differently phrased for participants who currently have Covid-19 (or suspect to have), who had had Covid-19 in the past (or suspected to had had) and people who had not had Covid-19. For example, for the latter group, participants were asked: 'I would like you to think about what it would be like if you personally got Covid-19. How much, if at all, do you agree or disagree with the following statements? (1) The symptoms of Covid-19 would be easy to recognise, (2) Covid-19 would have major consequences for my life, (3) Covid-19 symptoms would last a long time, (4) You could get Covid-19 again, (5) There are actions you could take to influence how your body responds to having Covid-19, (6) Your Covid-19 would be cured with treatment that doctors or nurses provide, (7) You would spend time worrying about having Covid-19, (8) Having Covid-19 would make you feel anxious'. We performed a factor analysis to assess which items could be taken together to establish a value score, with a higher score reflecting more negative views on Covid-19. Items 2, 3, 4, 7 and 8 loaded on a single component in principal component analysis. We calculated the average of these five items as a total score for illness representation (range 1–4).

### Data Collection

Ipsos MORI administered a self-reported questionnaire by conducting telephone interviews using Computer Aided Telephone Interviewing (CATI). Interviewers from Ipsos MORI receive training and have significant experience in conducting interviews into sensitive topics including mental and general health.

### Statistical Methods

The data were analysed using SPSS version 25.0. For all variables, answers ‘I don’t know’ and ‘I prefer not to say’ were treated as missing values and, therefore, excluded from the analyses. Most data only had few missing values, which were managed by listwise deletion of cases in any given analyses.

*P* values of *p* < 0.05 were taken as statistically significant. The Pearson bivariate correlation was used to explore the potential associations among variables. First, in simple regression analyses, we tested, hypothesis 1 , whether the sociodemographic groups associated with poorer mental health are still associated during the Covid-19 crisis and whether the social and psychological risk factors associated with mental health could be confirmed during the Covid-19 pandemic. Secondly, to address hypothesis 2, we tested whether the social and psychological risk factors exacerbated the effect in (some of) the sociodemographic groups. This was done through moderation model analyses with Hayes’ PROCESS macro (v 3.5, model 1) [48]. The SPSS macro PROCESS utilises a hierarchical multivariate regression analysis. The models were tested in two steps. For the moderation analysis, in a first step, one sociodemographic variable (age, gender or Scottish Index of Multiple Deprivation) was entered with one of the moderator variables (social support, relationship status, loneliness, perceived threat, illness representation). In the second step of the regression analyses, the interaction term between the moderator and the sociodemographic variable was entered. For the analyses, a 95% bias-corrected percentile bootstrapped confidence interval (CI) method was used, and 5,000 bootstrap re-samples were produced for moderation examination. Additionally, we employed conventional methods for plotting simple slopes to understand moderation effects, at one standard deviation below and above the mean [49].

### Ethical Approval

Ethical approval for this study was granted by the Life Sciences and Medicine College Ethics Review Board (CERB) at the University of Aberdeen (CERB/2020/5/1942).

## Results

### Participants

Participant characteristics are described in Table 1. The median age in years was 53, 61.4% of the sample were female, 3.8% lived in the most socioeconomically deprived areas and 15.6% lived in the most affluent areas, 62.9% were married or living together and 37.1% were single, widowed, divorced or separated. The means and standard deviations for sociodemographic variables are available in a supplementary file. The median for social support, loneliness, perceived threat and illness representation was 3.60 (range 1–4), 2.00 (range 1–5), 6.00 (range 1–16) and 3.00 (range 1–4), respectively. The percentage of people at risk of anxiety was 13.8%, with 12.3% being at risk of depression. Few people (0.6%) believed that they currently had Covid-19, 12.2% that they had had Covid-19 and 84.4% that they had not had Covid-19.

**Table 1:** Characteristics of people who participated in the CHARIS study (*N* = 1006)

		Scale range	*N*	%/^a^
Age (in years)	Median, IQR^a^		53	(34–65)^a^
Gender^b^	Male		388	38.6
	Female		616	61.4
Scottish Index of Multiple Deprivation^a^	1 (10% most deprived)		34	3.8
	2		60	6.8
	3		59	6.7
	4		68	7.7
	5		86	9.7
	6		103	11.6
	7		106	12.0
	8		123	13.9
	9		110	12.4
	10 (10% least deprived)		138	15.6
Social support	Median, IQR	1–4	3.60	(3.20- 4.00)^a^
Relationship status	Married/living together		631	62.9
	Single/Widowed/divorced/separated		372	37.1
Loneliness	Median, IQR	1–5	2.00	(1.33–2.67)^a^
Threat perception	Median, IQR	1–16	6.00	(4.00–8.00)^a^
Illness representation	Median, IQR	1–4	3.00	(2.60–3.50)^a^
Anxiety^b^	Normal: Lower than 3	0–6	865	86.2
	Probable case: 3 or higher		138	13.8
Depression^b^	Normal: Lower than 3	0–6	881	87.7
	Probable case: 3 or higher		123	12.3
Covid-19 status	Currently (think) you have		6	0.6
	(Think) you had had		123	12.2
	(Think) you had not had		849	84.4
	Don’t know		28	2.8

^a^IQR interquartile range

^b^Total numbers do not add up to 1006 due to missing data, for information on the number of missing values, see Table 2

Before testing the hypotheses, we examined the means, standard deviation and Pearson bivariate correlation among the study variables, as seen in Table 2. Results in Table 2 show that anxiety and depression were negatively associated with social support (i.e. higher rates of anxiety and depression associated with low social support) and positively related to loneliness, perceived threat and illness representations (i.e. higher rates anxiety and depression associated with higher rates of loneliness, perceived threat and more negative illness (Covid-19) representations). Age, gender and socioeconomic area deprivation were negatively associated with anxiety and depression (i.e. older age, male and more affluent areas associated with lower rates of anxiety and depression). The results reflect a preliminary analysis of the hypothesised associations which were as expected.

**Table 2:** Means, standard deviations and Pearson bivariate correlations of the study variables

	1	2	3	4	5	6	7	8	9	10
1. Anxiety										
2. Depression	.576***									
3. Relationship status	−.129***	−.215***								
4. Social support	−.149***	−.210***	.279***							
5. Loneliness	.391***	.466***	−.438***	−.287***						
6. Threat perception	.132**	.085*	.020	.089*	.071					
7. Illness representations	.195***	.130****	−.066*	.025	.179***	.399***				
8. Gender [0 = female, 1 = male]	−.195***	−.086***	.065*	.039	− .045	− .009	− .113***			
9. Age	−.219***	−.135***	−.185***	.265***	− .034	.093*	.130***	.003		
10.SIMD	−.086*	−.135***	.057	.058	− .091**	− .023	− .062	.033	.032	
*N*	1003	1004	1006	1003	1006	763	977	1004	995	887
Missing	3	2	-	3	-	243	29	2	11	119
Means	1.01	.928		3.49	2.15	6.40	2.99		50.96	6.48
Standard deviation	1.56	1.379		.597	.916	3.08	0.696		17.42	2.66
Internal consistency*	*r *= .70***	*r* = .50***	-	*α* = .62	*α* = .78	+	*α* = .81			

^*^Internal consistency reported reflect Cronbach’s alpha for scales with 3 or more items, correlation Pearson’s *r* for scales with 2 items

+ Theoretically, the construct of risk perception and severity do not need to be correlated

### Demographic Variables Associated with Anxiety and Depression

Table 3 shows the results of simple linear regression of sociodemographic factors (age, gender and Scottish Index of Multiple Deprivation (SIMD)) on to each of anxiety and depression. Age and gender accounted for a larger proportion of the variance in anxiety than in depression, whereas SIMD accounted for a larger proportion of the variance in depression. That said, none of the sociodemographic variables accounted for more than 5% of the variance in anxiety and 2% of the variance in depression.

**Table 3:** Simple linear regression of anxiety and depression on each of age, gender and SIMD

	*p* ≤	Beta (unstandardized)	*R*^2^
Anxiety			
Age	0.001	−0.20	0.048
Gender	0.001	−0.63	0.038
SIMD	0.02	−0.05	0.006
Depression			
Age	0.001	−0.01	0.18
Gender	0.01	−0.25	0.0007
SIMD	0.001	0.07	0.018

Gender: male = 1

### Moderators of the Relationship Between Sociodemographic Factors and Anxiety and Depression

Table 4 and Table 5 summarise the significant moderator analyses for anxiety and depression. Figures showing the follow-up simple slope analysis for each moderator analysis can be found in the supplementary file.

**Table 4:** Moderator analyses of the relationship between sociodemographic factors and anxiety

Factor	Moderator	*F*	*R*^2^	Δ*R*^2^	Beta for simple slope analyses		
Gender					Male		Female
	Loneliness	(1.997) 4.12^*^	0.019	0.003	0.51		0.72
	Illness reps	(1.968) 11.37^***^	0.078	0.011	0.10		0.58
Age					Young^a^	mean	Old^b^
	Loneliness	(1.988) 15.92^***^	0.207	0.013	0.86	0.67	0.47
	Illness reps	(1.959) 6.31^*^	0.102	0.006	0.68	0.51	0.33
SIMD					More deprived^a^	mean	Less deprived^b^
	Illness reps	(1.856) 8,62^**^	0.059	0.010	0.70	0.47	0.24
	Threat perception	(1.677) 4.45^*^	0.030	0.006	0.11	0.07	0.02
					Not married/living together		Married/living together
	Relationship status	(1.881) 5.75^*^	0.041	0.006	0.02		−0.08

^a^Age = 1SD below mean age, SIMD = 1SD below deprivation mean (more deprived)

^b^Age = 1SD above the mean age; 1SD above deprivation mean (less deprived)

^*^p ≤ 0.05; ^**^p ≤ 0.01; ^***^p ≤ 0.001

**Table 5:** Moderator analyses of the relationship between sociodemographic factors and depression

Factor	Moderator	*F*	*R*^2^	Δ*R*^2^	Beta for simple slope analyses		
Age					Young^a^	Mean	Old^b^
	Loneliness	(1,989) 8.76^**^	0.241	0.007	0.84	0.71	0.58
SIMD					more deprived^a^	mean	less deprived^b^
	Illness reps	(1,857) 5.36^*^	0.042	0.006	0.42	0.25	0.09

^a^Age = 1SD below mean age, SIMD = 1SD below deprivation mean (more deprived)

^b^Age = 1SD above the mean age; 1SD above deprivation mean (less deprived)

^*^p ≤ 0.05; ^**^p ≤ 0.01; ^***^p ≤ 0.001

For anxiety (Table 4), loneliness moderated the relationship between gender and anxiety and the positive association was more pronounced in females than males. Illness representations also moderated the relationship between gender and anxiety and the positive association was significant for females but not males. Loneliness moderated the relationship between age and anxiety and the positive association was more pronounced among younger people than older people. Illness representations moderated the relationship between age and anxiety and the positive association was more pronounced in younger compared with older people. Illness representations moderated the relationship between deprivation and anxiety and the positive association was more pronounced in deprived compared with affluent areas. Perceived threat moderated the relationship between deprivation and anxiety and the positive association was more pronounced in deprived compared with affluent areas. Relationship status moderated the relationship between deprivation and anxiety, and for those married/living together, there was a negative association compared with a positive association to those who were single/widowed/divorced/separated.

For depression (Table 5), loneliness moderated the relationship between age and depression and the positive association was more pronounced in younger than older people. Illness representations moderated the relationship between deprivation and depression and the positive association was more pronounced in deprived compared with affluent areas with those in more affluent areas not being affected by illness representations.

## Discussion

### Key Results

Overall, 13.8% and 12.3% of participants showed levels of anxiety and depression that met the threshold for likely anxiety and depression. The descriptive data and the results of the simple linear regression analyses show direct associations between anxiety and depression: sociodemographic variables (age, gender, socioeconomic area deprivation), social factors (social support, relationship status, loneliness) and psychological factors (perceived threat, illness representation). Social support (relationship status) moderated the relationship between socioeconomic area deprivation and anxiety; loneliness moderated the relationship between age and anxiety and depression, and gender and anxiety; perceived threat moderated the relationship between socioeconomic area deprivation and anxiety; illness representation moderated the relationship between age, gender, socioeconomic area deprivation and anxiety and between socioeconomic area deprivation and depression. The moderation effect was more pronounced in young adults, women and people living in the most deprived areas. Although these associations were statistically significant, they explained only a very small proportion of the variance in mental health.

### Consistency with Other Studies

The Scottish Health Survey which uses the General Health Questionnaire to measure mental distress and mental ill-health shows that 19% of adults had poor mental health which may include anxiety and depression [40]. Hence, the lower percentages of people showing levels of anxiety and depression in the current data may be due to the narrower focus on specific types of mental health problems. This data, from the first 2 weeks following the easing of lockdown restrictions in Scotland, confirm that younger people, women and people living in more socioeconomically deprived communities report higher levels of anxiety. These sociodemographic factors have been consistently reported as being associated with mental health and are long-standing intractable structural health inequalities, which means that they may be difficult (for reasons not investigated in this study) to quickly address during the current Covid-19 crisis. However, this study suggests that there are modifiable social and psychological factors that may be more readily addressed to improve mental health during the current crisis and potential future pandemics.

### Possible Explanations

#### Social Factors

Loneliness moderated the relationship between age and anxiety and depression, and between gender and anxiety. The positive association between loneliness and anxiety was more pronounced among women compared with men and among younger adults compared with older adults. A previous study conducted prior to the current pandemic found that loneliness declines with age and is higher in women than men [50]. This study offers a potential explanation for these findings. It suggests that women and younger adults may have worse mental health due to loneliness while men’s and older adults’ mental health is less affected by loneliness. It suggests that some of the mechanisms that explain the relationship between age, gender and poor mental health during the Covid-19 crisis, such as loneliness, are unlikely to be peculiar to the Covid-19 crisis and may persist. The study also suggests that if women and young people experience loneliness during the Covid-19 pandemic, then this is more likely to have a detrimental impact on their mental health than if men and older people experience loneliness. Public health policy-makers may therefore wish to consider addressing the problem of loneliness during pandemics through interventions delivered for instance, online [51] and target those groups of the population most at risk of experiencing loneliness.

#### Psychological Factors: Perceived Threat

Explanations for worse mental health in people who are socioeconomically disadvantaged have highlighted psychological mechanisms to explain this association [52]. As we have already highlighted in the introduction, a growing number of studies about Covid-19 have utilised the concept threat perception to understand the effects of the pandemic on mental health [19, 29–31]. These studies show that different dimensions of the construct threat perception (likelihood of contracting Covid-19, severity, control over getting infected, consequences of Covid-19) are associated with mental health. Our study also suggests that perceived threat (perceived severity and vulnerability) is directly associated with anxiety and depression. Threat appraisals may lead to anxiety or alternatively, anxiety may amplify perceptions of threat. There is ample evidence in experimental and clinical studies that people high in anxiety attend more to threatening stimuli and so may appraise the threat to be greater [53, 54]. In this study, perceived threat not only predicted anxiety, it also moderated the relationship between socioeconomic area deprivation and anxiety: anxiety was more pronounced among people in socioeconomically deprived areas as compared with people in affluent areas especially if they perceived the threat from Covid-19 to be greater. In the context of the Covid-19 pandemic, people living in the most socioeconomically deprived areas may perceive that they are at greater risk from Covid-19 and this perception may exacerbate anxiety. Their perception of being at greater risk has foundation; the UK Office for National Statistics data show a higher death rate in poorer areas [55]. In Wales for instance, the most deprived areas had a mortality rate for deaths involving Covid-19 of 44.6 deaths per 100,000 population, almost twice as high as the least deprived area of 23.2 deaths per 100,000 population [55].

This study found that women were more anxious and depressed than men but did not find that threat appraisal moderated the relationship between gender and anxiety and depression. Yet men, despite being objectively at greater risk of dying from Covid-19 compared with women [56], generally display lower threat perception of Covid-19 than women [57]. Public health agencies may wish to consider intervening to reduce threat perception during a pandemic when risk of infection is low in order to reduce anxiety but perhaps it would be inappropriate to reduce threat perception when risk of infection is high despite known associations with worse mental health.

#### Psychological Factors: Illness Representation

Illness representation was associated with anxiety and depression. Our findings are consistent with the substantial body of empirical work that is based on the CS-SRM [58] and with the CS-SRM as theoretically conceived [37]. According to the CS-SRM, an experience of a health threat initiates an internal process which aims to understand and decide how to manage the health threat and also regulate associated negative emotions [59]. This is a self-regulation process, which evolves over time as the health threat progresses [59], and therefore potentially provides opportunities for intervention to shift people’s illness representations (e.g. of Covid-19) and in doing so, improve mental health. The majority of research that draws on CS-SRM has focussed on illness representations in people living with chronic illness [58]. Like our study, previous research has examined relationships between cognitive (illness) representations based on people’s perceptions and beliefs about a health threat, which includes perceptual experiences of illness (e.g. symptoms, change in functioning) and abstract concepts and labels of an illness and health outcomes, including mental health [58]. A systematic review of illness perception and depression in patients with chronic kidney disease for instance found four studies which showed a relationship between illness perception and depression [60].

Illness representation was the most consistent moderator of the sociodemographic effect, moderating the effects of age, gender, socioeconomic area deprivation and anxiety and between socioeconomic area deprivation and depression. Hence, this study suggests that how people think about the illness Covid-19 is not only associated with mental health, but is also more pronounced in those groups of the population who are likely to experience worse mental health, i.e. younger people, women and people living the most socioeconomically deprived areas. Given that the CS-SRM recognises that people activate two cognitive systems in response to a health threat—one involving cognitive processes for regulating the objective health threat and the other involving emotional processes for regulating anxiety and fear [61], it is the latter processing which may be important for understanding the effects of Covid-19 on mental health. Several studies have shown that it is possible to modify illness representations with beneficial effects on health and healthcare outcomes [62, 63]. On the other hand, as discussed above, anxiety may draw the person’s attention to threatening aspects of the illness.

#### Limitations

The study has some limitations. First, this study only reports 2 weeks of the Covid-19 crisis and uses a cross-sectional design and therefore is unable to reveal the extent to which anxiety and depression and associated moderating psychological and social factors change over the course of the pandemic or establish causality. Second, measures of anxiety and depression rely on self-report which is open to sources of bias, such as men being less willing than women to report poor mental health [64]. Nonetheless, participants self-reported on mental health and other variables for the previous week and we therefore surmise that recall bias is minimal in this study. Third, the telephone survey was restricted to 15 min duration which meant that only brief measures of variables were used and not all were validated. Fourth, while the social and psychological variables (social support, relationship status, loneliness, threat perception, illness representations) were carefully chosen based on strong empirical evidence and theorised models, mental health during the Covid-19 crisis is likely to be moderated by a range of distal and proximal determinants. This is possibly the main reason why this study found that only very small percentages of variation in anxiety and depression could be explained by social and psychological factors; for example, only 4.8% of the variation in anxiety could be explained by the model containing only age. Thus, future investigations about mental health during the Covid-19 pandemic should consider expanding upon this research. Finally, we did not have information about participants’ mental health prior to the Covid-19 pandemic nor did we collect information about any psychiatric diagnosis or psychiatric medication which means that we are only able to assess anxiety and depression by the PHQ-4 which is a validated screening tool for potential anxiety and depression [42].

## Conclusions

Previous mental health inequalities seem durable and persist in the current Covid-19 crisis. This study shows, for instance, that younger adults, women and people living in the most deprived areas have greater anxiety and depression. Similarly, some psychological and social mechanisms including loneliness, threat perception and illness representations that explain some of the variation in mental health in the adult population prior to the current Covid-19 crisis also have relevance during the crisis. Importantly, each of these social and psychological factors also appears to exaggerate the effect of sociodemographic factors, offering some explanation and possible opportunities for ameliorating these effects during pandemics.

## Electronic supplementary material

Below is the link to the electronic supplementary material.

Supplementary file1 (DOCX 90 KB)

Supplementary file2 (DOCX 15 KB)

## Data Availability

The data that support the findings of this study are available on request from the corresponding author. The data are not currently publicly available due to the research team still publishing from these data.
