# Supplementary material for: Sociodemographic and Psychological Risk Factors for Anxiety and Depression: Findings from the Covid-19 Health and Adherence Research in Scotland on Mental Health (CHARIS-MH) Cross-sectional Survey
Source: Int J Behav Med. 2021 Mar 3;28(6):788–800. doi: 10.1007/s12529-021-09967-z (PMC7929550; doi:10.1007/s12529-021-09967-z)
Supplement: Supplementary file 1 — Supplementary file1 (DOCX 90 KB) [file 12529_2021_9967_MOESM1_ESM.docx]

**Supplementary File: Simple slope analysis for each moderator analysis**

Figure 1: Loneliness for gender and anxiety


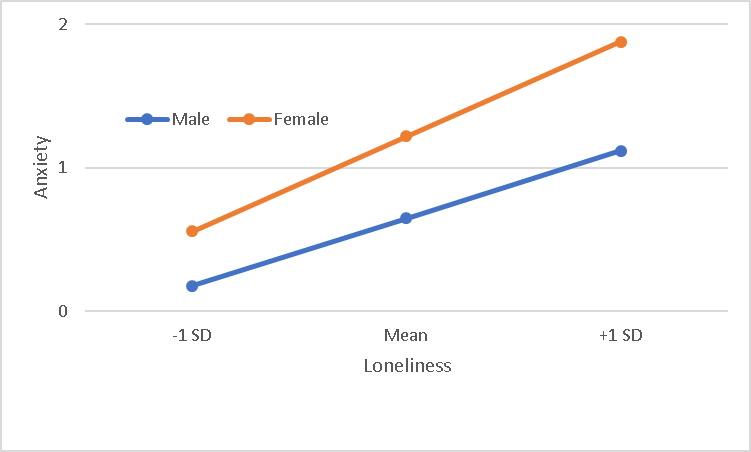


Figure 1: Illness representations for gender and anxiety


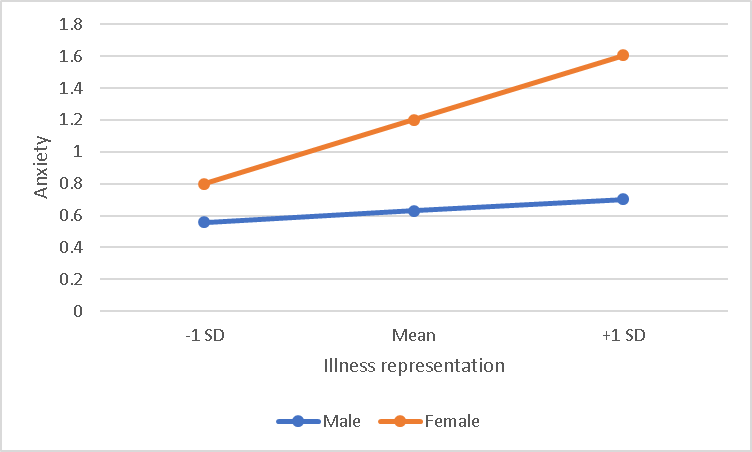


Figure 3: Loneliness for age and anxiety


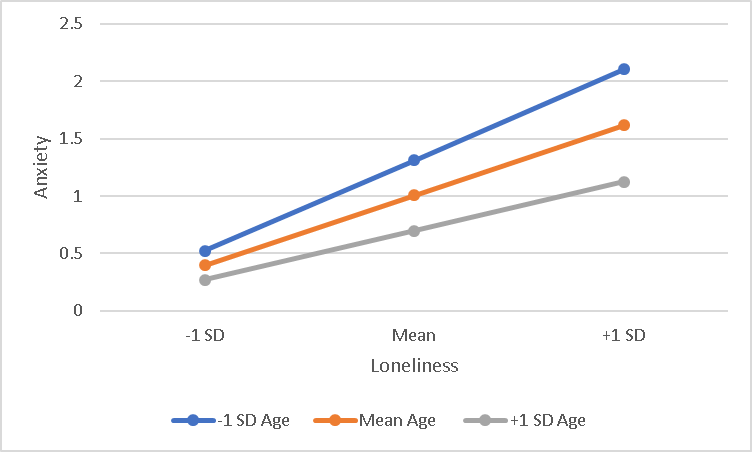


Figure 4: Illness representations for age and anxiety


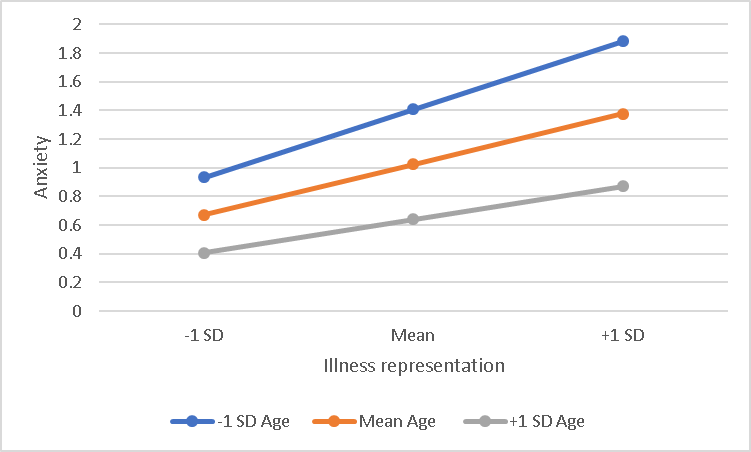


Figure 5: Illness representations for deprivation and anxiety


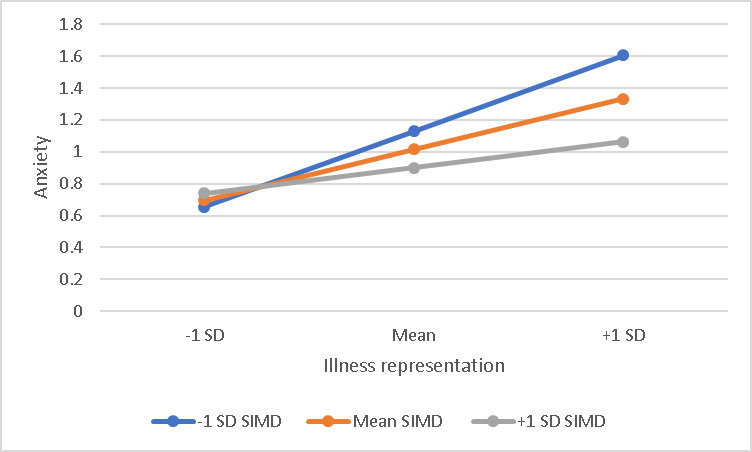


Figure 6: Perceived threat for deprivation and anxiety


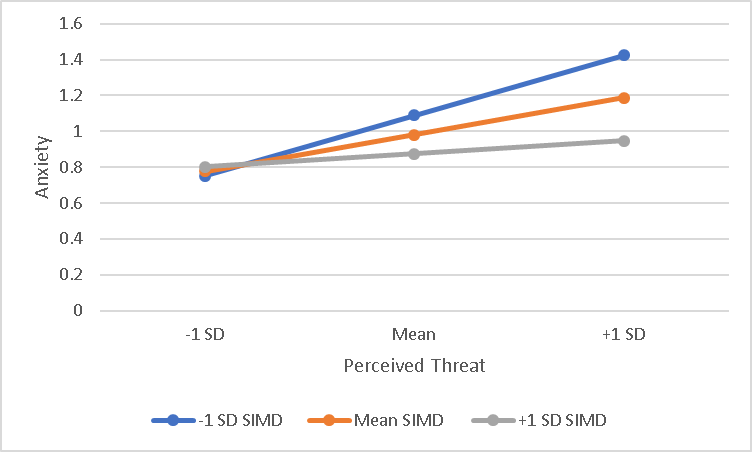


Figure 7: Relationship status for anxiety and deprivation


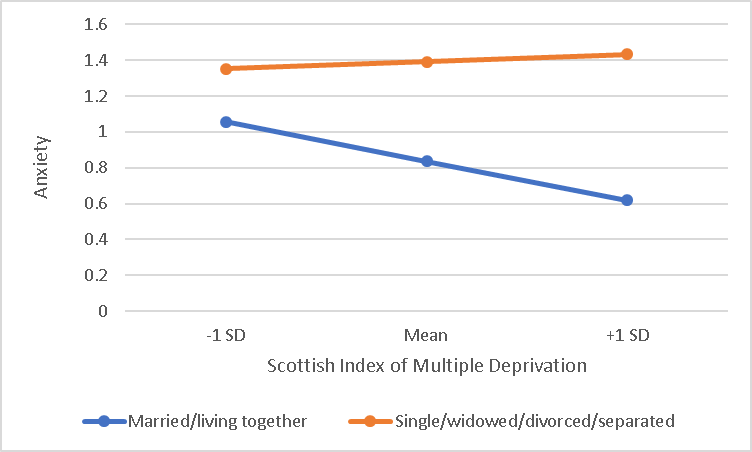


Figure 8: Loneliness for age and depression


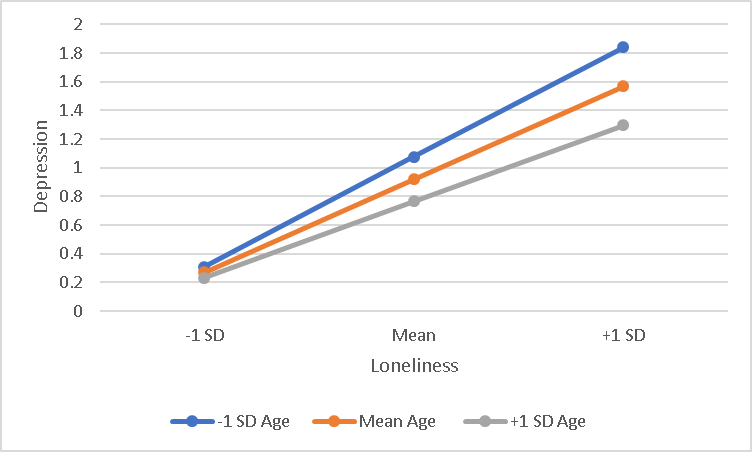


Figure 9: Illness representations for deprivation and depression

**
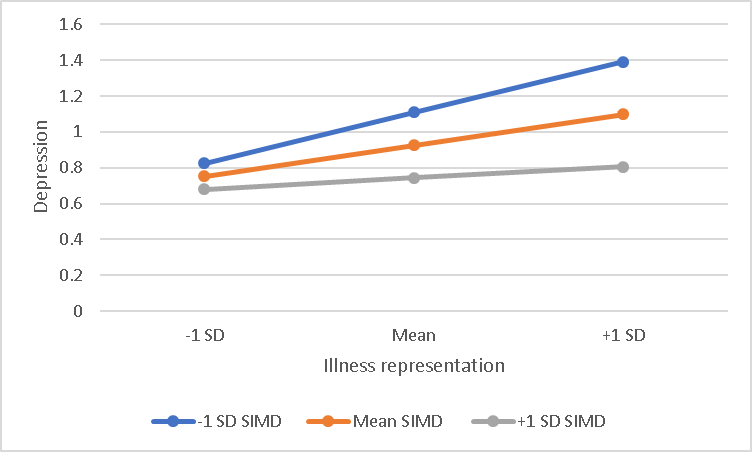
**
