# Supplementary material for: Sociodemographic and Psychological Risk Factors for Anxiety and Depression: Findings from the Covid-19 Health and Adherence Research in Scotland on Mental Health (CHARIS-MH) Cross-sectional Survey
Source: Int J Behav Med. 2021 Mar 3;28(6):788–800. doi: 10.1007/s12529-021-09967-z (PMC7929550; doi:10.1007/s12529-021-09967-z)
Supplement: Supplementary file 2 — Supplementary file2 (DOCX 15 KB) [file 12529_2021_9967_MOESM2_ESM.docx]

The Means and Standard Deviations on Anxiety and Depression for the sociodemographic groups.

|  |  | **Anxiety** |  | **Depression** |  |
| --- | --- | --- | --- | --- | --- |
|  |  | **Mean** | **SD** | **Mean** | **SD** |
| **Age** | 16-24 | 1.73 | 1.97 | 1.51 | 1.49 |
|  | 25-34 | 1.21 | 1.67 | 1.02 | 1.39 |
|  | 35-44 | 1.54 | 1.90 | 1.27 | 1.71 |
|  | 45-54 | 1.00 | 1.59 | 0.76 | 1.32 |
|  | 55-64 | 0.70 | 1.21 | 0.78 | 1.23 |
|  | 65+ | 0.67 | 1.26 | 0.78 | 1.30 |
| **Gender** | Female | 1.24 | 1.71 | 1.02 | 1.43 |
|  | Male | 0.62 | 1.18 | 0.77 | 1.27 |
| Scottish Index of Multiple Deprivationa | 1 (10% most deprived) | 1.21 | 1.72 | 1.24 | 1.94 |
|  | 2 | 1.30 | 1.93 | 1.42 | 1.62 |
|  | 3 | 1.57 | 1.86 | 1.42 | 1.52 |
|  | 4 | 1.03 | 1.78 | 0.96 | 1.62 |
|  | 5 | 0.92 | 1.57 | 0.92 | 1.43 |
|  | 6 | 0.95 | 1.52 | 0.72 | 1.26 |
|  | 7 | 1.18 | 1.71 | 1.13 | 1.36 |
|  | 8 | 0.91 | 1.38 | 0.83 | 1.36 |
|  | 9 | 0.86 | 1.35 | 0.82 | 1.31 |
|  | 10 (10% least deprived) | 0.90 | 1.45 | 0.67 | 1.09 |
